# Reinforcement learning of biomimetic navigation: a model problem for sperm chemotaxis

**DOI:** 10.1140/epje/s10189-024-00451-6

**Published:** 2024-09-27

**Authors:** Omar Mohamed, Alan C. H. Tsang

**Affiliations:** Department of Mechanical Engineering, The University of Hong Kong, Pokfulam Road, Pok Fu Lam, Hong Kong China

## Abstract

**Abstract:**

Motile biological cells can respond to local environmental cues and exhibit various navigation strategies to search for specific targets. These navigation strategies usually involve tuning of key biophysical parameters of the cells, such that the cells can modulate their trajectories to move in response to the detected signals. Here we introduce a reinforcement learning approach to modulate key biophysical parameters and realize navigation strategies reminiscent to those developed by biological cells. We present this approach using sperm chemotaxis toward an egg as a paradigm. By modulating the trajectory curvature of a sperm cell model, the navigation strategies informed by reinforcement learning are capable to resemble sperm chemotaxis observed in experiments. This approach provides an alternative method to capture biologically relevant navigation strategies, which may inform the necessary parameter modulations required for obtaining specific navigation strategies and guide the design of biomimetic micro-robotics.

**Graphical abstract:**

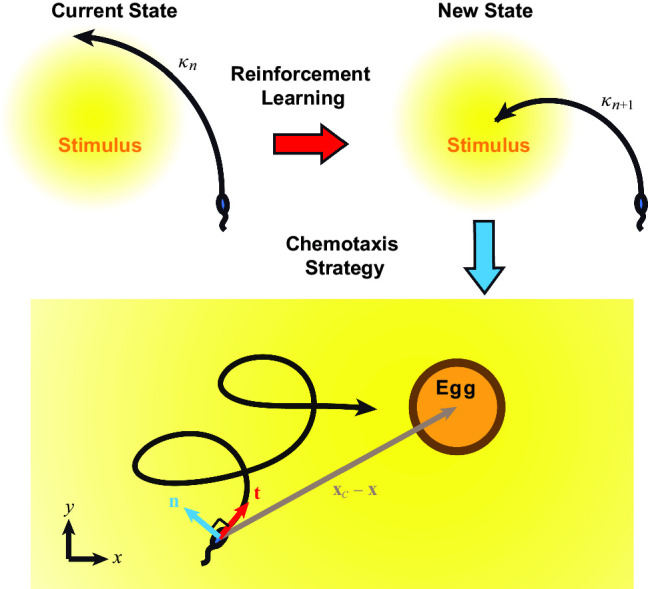

## Introduction

Motile biological cells respond to environmental cues such as chemicals and light to modulate their moving trajectories, leading to effective navigation strategies for exploring the environment [1, 2]. For example, sperm cells modulate the curvature of their helical trajectories in response to the local chemical field to move up the chemical gradient and bias their paths toward the egg [3–6]. Bacteria exhibit run-and-tumble to navigate the surrounding chemical field and detect food molecules [7–9]. Motile algae and other pond dwellers swim in response to light and optimize the light condition for photosynthesis [10–15]. The success of these navigation strategies relies on the modulation of specific key biophysical parameters (e.g., path curvature in sperm cells, tumbling rate in bacteria, reorientation rate in motile algae) in response to environmental cues. Various biophysical models have been proposed to explain the underlying mechanisms of various navigation strategies of motile biological cells [9–11, 13, 15, 16]. Here we consider an alternative method of finding biologically relevant navigation strategies by reinforcement learning. We ask general questions of whether and how reinforcement learning can enable biomimetic navigation strategies by tuning key biophysical parameters.

Recent surge in artificial intelligence has sparked new research directions in biophysical problems and the design of bio-inspired robotics [17–20]. The vast potential of machine learning approaches have been demonstrated in obtaining effective locomotion strategies for bio-inspired micro-robotics [21–24]. These machine learning approaches can be applied to complex navigation problems that involve hydrodynamics flows, thermal fluctuations, obstacles and chemical fields. For example, reinforcement learning enables micro-swimmers to navigate toward a specific target direction [25–27], avoiding from being trapped by local vortical flows [28, 29], and searching for local maxima in chemical fields [30–33]. These approaches can also be extended to navigation problems involving multiple swimmers such as pursuit-evasion and schooling [34, 35]. Recently, artificial micro-swimmers with control systems integrated with reinforcement learning algorithms are realized experimentally [20, 36, 37].

In this work, we present a reinforcement learning approach for biomimetic navigation. We demonstrate this approach using a model problem of sperm chemotaxis toward an egg as a generic example. Reinforcement learning provides an effective policy to modulate the path curvature of the sperm model in response to the local chemical signal, so that the sperm can steer its path to reach the egg eventually. By improving the policy over learning episodes, the model eventually exhibits navigation strategies that are reminiscent to sperm chemotaxis observed experimentally.

**Fig. 1:**
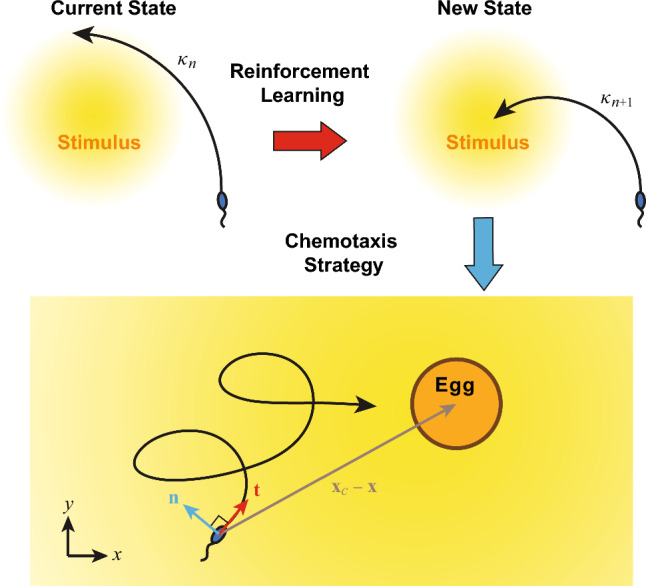
Reinforcement learning of sperm cell chemotaxis. Our reinforcement learning algorithm determines how the sperm cell modulate its path curvature in response to detected chemical field. This will result in an effective navigation strategy that guides the sperm cell toward the egg. The blue and red arrows indicate the swimming direction $\textbf{t}$ and the orthogonal direction $\textbf{n}$, respectively. The gray arrows represent the relative distance between the egg and the sperm cell

### Dynamic model of sperm cell

We start by introducing the sperm cell model for the navigation problem (Fig. 1). Following the model proposed by Friedrich and Jülicher [16], the dynamics of a sperm cell can be described by the Frenet-Serret equations. Here we account for a reduced 2D model, which can be considered as a sperm cell swimming on a 2D plane perpendicular to a surface. An extension of the model to 3D will be discussed in a later section. The swimming path $\textbf{x}(t)$, which captures the position of the sperm head averaged over a flagella beating cycle, is governed by the following equations:

1 $$\begin{aligned} \begin{aligned} \dot{\textbf{x}}=v\textbf{t}, \\ \dot{\textbf{t}}=v\kappa \textbf{n}, \\ \dot{\textbf{n}}=-v\kappa \textbf{t}. \end{aligned} \end{aligned}$$

Here, *v* is the swimming speed and $\kappa$ is the local curvature of the swimming path. The instantaneous swimming direction of the sperm cell is denoted by a unit vector $\textbf{t}$. The unit vector $\textbf{n}$ is orthogonal to $\textbf{t}$. In this model, when the sperm cell swims in a path with a constant $\kappa$, it will trace out a circular trajectory with a radius of $1/\kappa$.

The sperm cell can detect a concentration stimulus $c(\textbf{x}(t))$ due to the chemoattractant released by the egg. Here we consider a radially decaying chemical concentration field due to the chemoattractant:

2 $$\begin{aligned} \begin{aligned} c(\textbf{x}(t))=\frac{c_0}{|\textbf{x}(t)-\textbf{x}_c|}, \end{aligned} \end{aligned}$$

where $c_0$ is the strength of the chemical field and $\textbf{x}_c$ is the location of the egg’s center.

Model parameters can be estimated from the experimental data of sperm chemotaxis in previous works [6, 38, 39], i.e., $v \sim$ 100–200 $\upmu$m/s and $\kappa \sim$ 0.01–0.05 $\upmu \textrm{m}^{-1}$.

Friedrich and Jülicher proposed a set of ingenious stimulus–response functions to describe how the sperm cell responds to the local chemical field and modulates its $\kappa$ (i.e., $\kappa =\kappa _0+\kappa _1(a(t)-1)$) to achieve chemotaxis [16]: $\eta \dot{a}=pc-a; \mu \dot{p}=1-a.$ The adaptation variable *p* couples with the variable *a* to modulate $\kappa$ in response to the local chemical field *c*. $\eta$ and $\mu$ are constants for tuning the relaxation time.

Here we employ an alternative approach of sperm chemotaxis by reinforcement learning: instead of following an explicit stimulus–response function to steer its trajectory, our reinforcement learning algorithm determines how the sperm cell modulates its path curvature in response to the detected chemical field to achieve effective chemotaxis strategies.

### Reinforcement learning

We employ the standard *Q*-learning algorithm to obtain an effective policy to modulate the path curvature for chemotactic navigation [40]. The reinforcement learning algorithm evaluates the best action to be taken by the sperm cell at a particular learning step to move up the chemical gradient. Namely, the sperm cell follows the swimming dynamics given by Eq. (1), while $\kappa$ is adjusted according to the action informed by the reinforcement learning algorithm.

Here we outline the state, the action and the reward required to set up the reinforcement learning algorithm. At a given learning step *n*, the sperm cell can sense the local chemical field $c_n=c(\textbf{x}(t_{n}))$. We assume that the sperm cell has a short-term memory about the detected chemical field and it can determine whether there is an increase or a decrease in the detected chemical field due to its motion in the current learning step, i.e., $\Delta c_n=c_n-c_{n-1}>0$ or $\Delta c_n=c_n-c_{n-1}<0$. A short-term memory of detected stimuli has been observed in bacterial cells and eukaryotic cells [41, 42]. The size of a learning step is given by $\delta t = t_{n}-t_{n-1}$. We note that Eq. (1) are solved numerically with a much smaller time step compared to $\delta t$ for better accuracy. The state $s_n$ of the reinforcement learning agent is specified by the sign for the change in the local chemical field $sgn(\Delta c_n)$ and the current path curvature $\kappa _n$. Here $\kappa _n$ is mapped into a set of $L=2X+1$ discrete states with the interval $[\kappa _0-X \delta \kappa , \kappa _0+X \delta \kappa ]$, where $\kappa _0$ is the initial value for $\kappa$ at $n=0$ and $\delta \kappa$ is the difference in values of $\kappa$ between two consecutive states. The parameter *X* determines the range of $\kappa$ considered in the learning model. Here we choose *X* to be large enough such that $\kappa$ in our simulations do not reach the maximum or minimum value of the considered range. The sperm cell can perform an action $a_n$ to modify the original path curvature from $\kappa _n$ to a new path curvature $\kappa _{n+1}$ to modulate its trajectory (Fig. 1). The set of actions include increasing $\kappa _n$ by $\delta \kappa$, decreasing $\kappa _n$ by $\delta \kappa$, and keeping $\kappa _n$ unchanged. The effectiveness of the action is measured by a reward that accounts for the increase in local concentration field due to the action, i.e., $r_n=\beta (1/c_{n}-1/c_{n+1})$, where $\beta$ is a weighting factor for the reward. We set $\beta =1/c_0$, unless otherwise specified. A reciprocal function of the local chemical field is used to define the reward to avoid singularity problem at the origin of the egg, i.e., $c \rightarrow \infty$ when $|\textbf{x}(t)-\textbf{x}_c| \rightarrow 0$ in Eq. 2. We note that other reward functions are possible if a regularized function of chemical field (e.g., Gaussian function) is considered. Here we choose a robust reward function with a relatively simple form.

An action-value function $Q(s_n, a_n)$ is introduced to quantify the expected long-term reward for taking the action $a_n$ given the state $s_n$. This *Q*-matrix encodes the adaptive decision-making intelligence of the reinforcement learning agent. After each learning step, the *Q*-matrix evolves based on the information exploited from the detected chemical concentration,

3 $$\begin{aligned}  &   Q(s_n,a_n) \leftarrow Q(s_n,a_n) \nonumber \\  &   \quad +\alpha \Big [ r_n + \gamma \max _{a_{n+1}} Q(s_{n+1},a_{n+1}) - Q(s_n,a_n)\Big ],\qquad \end{aligned}$$

where $\alpha$ is the learning rate $(0\le \alpha \le 1)$ that determines to what extent new information overrides old information. For a deterministic system, a larger $\alpha$ corresponds to a faster learning speed, hence we fixed $\alpha =1$ to maximize the learning speed. The discount factor $\gamma$ ($0<\gamma <1$) determines how much the future reward is accounted relative to the immediate reward. The reinforcement learning agent is shortsighted when $\gamma$ is small and tends to maximize the immediate reward; The reinforcement learning agent is farsighted when $\gamma$ is large and tends to maximize the long-term reward. We also implemented an $\epsilon$-greedy selection scheme to avoid being trapped in locally optimal policy. That is, in each learning step, the sperm cell has a probability $1-\epsilon$ to choose the best action recommended by the *Q*-matrix or has a probability $\epsilon$ to take a random action to explore other possible solutions. Unless otherwise specified, we set $\gamma =0.8$ and $\epsilon =0.1$ in all our simulations.

To further enhance the navigation performance of the cell, we break the learning session into multiple episodes. That is, we repeat the learning process and re-train the reinforcement learning agent again after each episode. In the new episode, we reuse the *Q*-matrix obtained at the end of the previous episode as a new initial condition, while keeping the other initial conditions unchanged. This allows us to bring the experience gained in the previous episode to the new episode. We divide the learning process into a total of $N_e$ episodes, with each episode consisting of $N_t$ learning steps. When sufficient episodes of learning are performed, the model will develop an effective navigation strategy for chemotaxis (Fig. 1).

## Results

### Sperm chemotaxis enabled by reinforcement learning

**Fig. 2:**
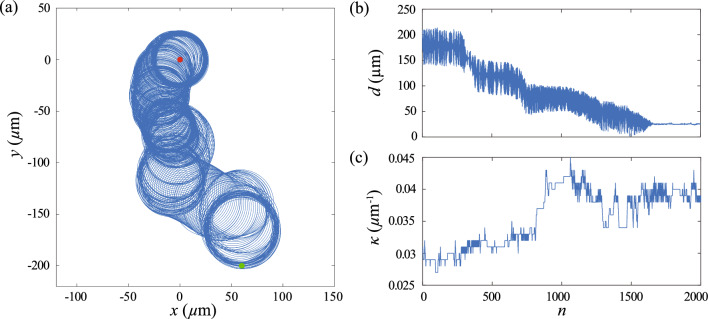
A typical example of reinforcement learning of a sperm model in 2D. **a** An example trajectory of sperm chemotaxis obtained by reinforcement learning. The green spot denotes the initial position of the cell and the red spot denotes the position of the egg. **b** Change in relative distance *d* between the cell and the egg over increased learning step. **c** Change in path curvature $\kappa$ over increased learning step. Parameters used: $\kappa _0=0.03$ $\upmu \textrm{m}^{-1}$, $\delta \kappa = 0.001$ $\upmu \textrm{m}^{-1}$, $\delta t =0.5$ s, $v=200$ $\upmu$m/s, $\textbf{x}(t=0)=(360\hat{x}-360\hat{y})$ $\upmu$m

We now consider the reinforcement learning of the 2D sperm cell model. Figure 2 depicts a typical learning process of the model. The sperm cell initially circles around and keeps exploring the surrounding chemical field (Fig. 2a), where the relative distance *d* between the sperm cell and the egg fluctuates periodically according to the size of the circular path (Fig. 2b) and the path curvature $\kappa$ remains more or less constant (Fig. 2c). After accumulating enough information about the chemical field, the sperm cell develops an effective navigation strategy based on the reinforcement learning algorithm to slowly steer its circular path toward the egg. The sperm cell eventually circles stably around the egg with a constant *d* at learning step $n>1700$ (Fig 2a, b). This chemotaxis strategy developed by reinforcement learning is reminiscent to what have been observed in experiments as well as other previous biophysical models of sperm cells [6, 16].

**Fig. 3:**
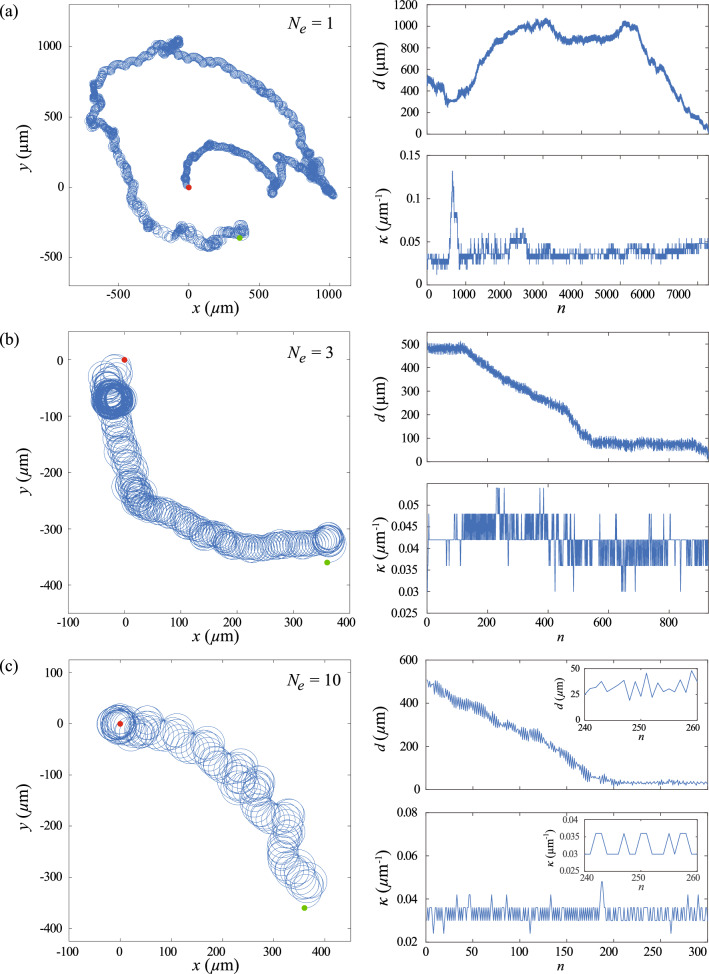
Episode learning enables progressive improvement in navigation strategies of the sperm cell model in 2D. Trajectory, relative distance *d* and variation in $\kappa$ of the cell at **a** $N_e=1$, **b** $N_e=3$, **c** $N_e=10$. Parameters used: $\kappa _0 =0.03$ $\upmu \textrm{m}^{-1}$, $\delta \kappa = 0.006$ $\upmu \textrm{m}^{-1}$, $\delta t =0.5$ s, $v=120$ $\upmu$m/s, $\textbf{x}(t=0)=(360\hat{x}-360\hat{y})$ $\upmu$m. The insets in (**c**) illustrate nearly periodic variations of *d* and $\kappa$ for the converged strategy when the cell reaches the egg and stably orbits around it

The performance of chemotactic navigation can be further improved via episode learning. Figure 3 depicts the continuous improvement in chemotaxis strategy over increased episodes. In the first episode where the sperm cell starts with a non-trained condition, the cell spends a very long time ($n>5000$, time equals to $n\delta t$) to navigate the chemical field and find the egg (Fig. 3a). In contrast, at increased episodes (i.e., $N_e=3$ & $N_e=10$), the sperm cell quickly steers its path toward the egg within much shorter times ($n<1000$) as shown in Fig. 3b,c. It is notable that the improved chemotaxis strategies obtained via episode learning display a nearly periodic oscillation in $\kappa$ (Fig. 3b, c). A similar periodic variation in $\kappa$ is also observed in the chemotaxis strategy based on a biophysical model with a stimulus–response function [16]. Here we obtain an equally effective chemotaxis strategy via reinforcement learning. We note that the learning result is independent of the initial position and the cell orientation. After sufficient learning steps, the cell will perform the same effective chemotaxis strategy even if the initial position and orientation are changed.

**Fig. 4:**
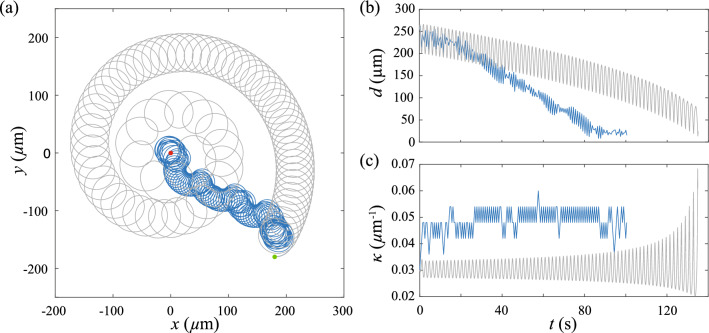
Comparison of the navigation strategies obtained by the reinforcement learning approach ($N_e=10$, blue colored lines) and the model by Friedrich and Jülicher (grey colored lines) [16]. Comparison of **a** trajectories, **b** relative distance *d* and **c** variation in $\kappa$. Parameters used: $\delta \kappa = 0.006$ $\upmu \textrm{m}^{-1}$, $\delta t =0.5$ s (reinforcement learning model); $\kappa _1=0.025$ $\upmu \textrm{m}^{-1}$, $\eta =0.14$ s, $\mu =0.725$ pM s^-1^ (model by Friedrich and Jülicher). For both cases, $v=120$ $\upmu$m/s, $\kappa _0 =0.03$ $\upmu \textrm{m}^{-1}$, $c_0=10$ pM, $\textbf{x}(t=0)=(180\hat{x}-180\hat{y})$ $\upmu$m

To benchmark the effectiveness of chemotactic navigation strategy obtained by the reinforcement learning approach, we quantitatively compare the navigation performance of its converged strategy ($N_e=10$) with the navigation strategy obtained from the stimulus–response model proposed by Friedrich and Jülicher (Fig. 4). The stimulus–response model displays a smooth and spiral trajectory toward the source, whereas the reinforcement learning model displays a curvy path with relatively larger curvature values (i.e., smaller open circular orbits) (Fig. 4a). Both strategies exhibit roughly periodic modulations of $\kappa$ and reach the source at similar times (Fig. 4b). We perform 10 sets of simulations with the same initial distance from the source but different initial orientation for each model. We measure the time required by the two strategies to approach the source with $d<50$ $\upmu$m. A preliminary comparison shows that the strategy given by the reinforcement learning model (101.9 ± 5 s; MEAN and SEM) is slightly fast than the strategy given by the stimulus–response model ($134.4 \pm 8.4$ s; MEAN and SEM) to reach the source. Nevertheless, both strategies capture the salient features of nearly periodic curvature modulation of sperm chemotaxis observed in experiments (Fig. 4c).

### Success rate of navigation

**Fig. 5:**
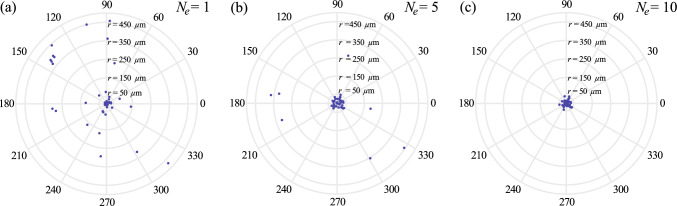
Scatter plots of the final positions of the 2D sperm cell in 50 trials at different number of episodes. A total of $n=50000$ learning steps are performed in each trial. The results at **a** $N_e=1$, **b** $N_e=5$ and **c** $N_e=10$ are displayed. $100\%$ successful rate is achieved at $N_e=10$. Parameters used: $\kappa _0 =0.03$ $\upmu \textrm{m}^{-1}$, $\delta \kappa =0.006$ $\upmu \textrm{m}^{-1}$, $\delta t =0.5$ s, $v=120$ $\upmu$m/s, $\textbf{x}(t=0)=(360\hat{x}-360\hat{y}+0\hat{z})$ $\upmu$m

We then investigate the success rate of the chemotaxis strategies obtained by reinforcement learning. The success rate of navigation is defined as the proportion of cells that successfully reach the egg within a time period $t_p$, where we set $t_p=25000$ s. By reaching the egg, we refer to the situation when the cell circles around the egg stably with a radius less than or equal to a certain threshold. To this end, we set the threshold to be $d \le 50$ $\upmu$m which agrees with the typical radius of an egg (i.e., 50 $\upmu$m) [43]. Although a 100% success rate cannot be achieved in a single episode of learning, the success rate of navigation can be improved significantly via episode learning. Figure 5 shows scattered plots of the final positions of the cells in 50 simulations at $N_e=1$ to $N_e=3$. At $N_e=1$, 20/50 of the cells reach the egg (Fig. 5a). At $N_e=5$, the success rate improves and 41/50 of the cells reach the egg (Fig. 5b). At $N_e=10$, all the cells reach the egg and a 100 % success rate is achieved (Fig. 5c). These results demonstrate how the reinforcement learning approach can achieve progressive improvement in chemotaxis strategies via episode learning.

**Fig. 6:**
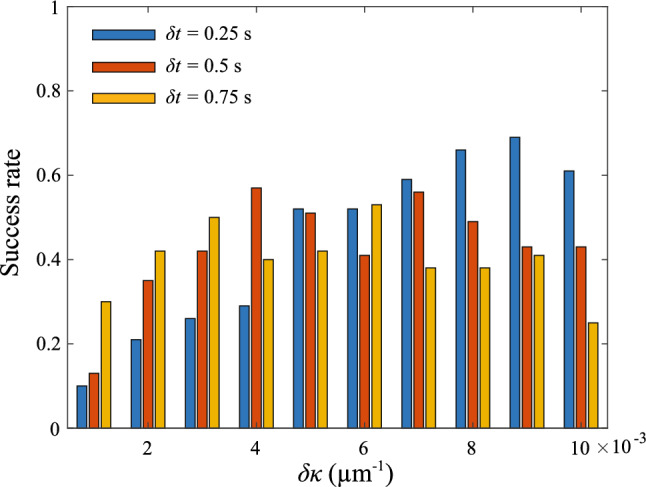
The success rate of navigation of a 2D sperm cell with different $\delta \kappa$ and $\delta t$ at $N_e=1$. Success rate for $\delta \kappa$ at 0.002 $\upmu \hbox {m}^{-1}$, 0.004 $\upmu \hbox {m}^{-1}$, 0.006 $\upmu \hbox {m}^{-1}$, 0.008 $\upmu \hbox {m}^{-1}$, 0.01 $\upmu \hbox {m}^{-1}$ and $\delta t$ at 0.25 s (represented by blue bars), 0.5 s (represented by red bars) and 0.75 s (represented by yellow bars) are considered. 100 simulations were performed for each case. Parameters used: $\kappa _0 =0.03$ $\upmu \hbox {m}^{-1}$, $v=120$ $\upmu$m/s, $\textbf{x}(t=0)=(360\hat{x}-360\hat{y}+0\hat{z})$ $\upmu$m

We also test the sensitivity of learning parameters on the success rate. We focus on the success rate at $N_e=1$ and perform a set of simulations with different $\delta \kappa$ and $\delta t$. We consider the cases with $\delta \kappa = 2 \times 10^{-3}$ $\upmu \hbox {m}^{-1}$, $4 \times 10^{-3}$ $\upmu \hbox {m}^{-1}$, $6 \times 10^{-3}$ $\upmu \hbox {m}^{-1}$, $8 \times 10^{-3}$ $\upmu \hbox {m}^{-1}$, $10 \times 10^{-3}$ $\upmu \hbox {m}^{-1}$ and $\delta t = 0.25$ s, 0.5 s, 0.75 s. We perform 100 simulations for each case, where the results are summarized in Fig. 6. For all considered values of $\delta t$, represented by different colored bars in Fig. 6, the success rate follows a non-monotonic change with increased $\delta \kappa$. Namely, we observe an increase in the success rate for a small increase in $\delta \kappa$ initially, which is then followed by a decrease in the success rate for a further increase in $\delta \kappa$. We note that a small $\delta t$ may not be beneficial for navigation as it takes time to observe the effect of an action, i.e., whether there is an increase or a decrease in the detected chemical concentration due to an action. A large learning step may not be beneficial either as the correlation between the action and the change in the detected chemical concentration will decay over time. This complexity in the choice of learning step results in the non-montonic behavior of success rate with increased $\delta t$ as shown in Fig. 6. The optimal value of $\delta \kappa$ for maximum success rate thus depends on the value of $\delta t$. Figure 6 provides guidance for appropriate choices of $\delta \kappa$ and $\delta t$.

### Robustness under noises

We then investigate the robustness of our reinforcement learning approach by incorporating the influence of signal noise and curvature noise into the sperm cell model [44, 45]. Following the coarse-grained model of stochastic swimming paths of sperm cells proposed by Friedrich and Jülicher [44], we assume that chemoattractant molecules bind to specific receptors on the flagellar membrane with a total binding rate of $q(t)=\lambda c(\mathbf {x(t)})$, where $\lambda$ is a binding constant. The mean time interval of a binding event 1/*q* is assumed to be large compared to the variation timescale of *q*(*t*), and 1/*q* is small compared to the relaxation time of the signaling module. In such a situation, the detected stimulus due to stochastic binding events can be represented by a coarse-grained model:

4 $$\begin{aligned} \begin{aligned} c_\text {noise}(\textbf{x}(t))=q(t)+\sqrt{q(t)}\xi _c(t), \end{aligned} \end{aligned}$$

where $\xi _c$ is a Gaussian noise with a normal distribution of zero mean and unit variance.

We also account for the curvature noise from the fluctuations of flagella beats due to stochastic activities of molecular motors inside the flagellum. This curvature noise is captured as:

5 $$\begin{aligned} \begin{aligned} \kappa _\text {noise}(t)= \bar{\kappa } (t)+\xi _\kappa . \end{aligned} \end{aligned}$$

where $\bar{\kappa }$ is the mean curvature and $\xi _\kappa$ is the curvature noise given by a Gaussian noise with a normal distribution of zero mean and variance $\sigma _\kappa$.

**Fig. 7:**
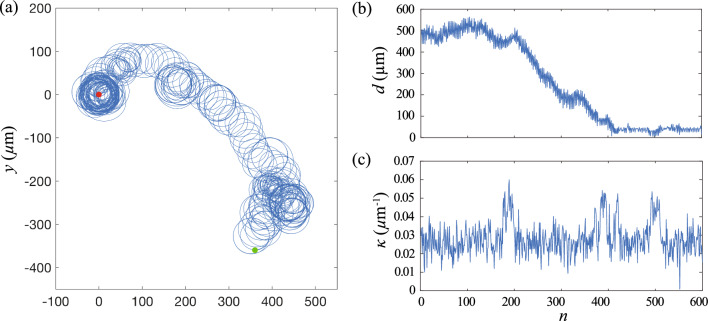
Chemotaxis of the sperm model under signal noise and curvature noise. **a** An example trajectory of sperm cell chemotaxis under noises after sufficient learning at $N_e=10$. **b** Change in relative distance *d* between the cell and the egg over increased learning step. **c** Change in curvature $\kappa$ of the cell over increased learning step. Parameters used: $\kappa _0=0.03$ $\upmu \hbox {m}^{-1}$, $\delta \kappa = 0.006$ $\upmu \hbox {m}^{-1}$, $\delta t =0.75$ s, $v=120$ $\upmu$m/s, $\textbf{x}(t=0)=(360\hat{x}-360\hat{y})$ $\upmu$m, $\lambda =10$ pM$^{-1}$ s$^{-1}$, $\sigma _\kappa =0.005$, $\beta =1/(c_0 \lambda )$

Figure 7a depicts an example trajectory of the sperm cell under signal and curvature noises after sufficient learning episodes (i.e., $N_e=10$). Compared to the trajectory without noises (Fig. 3c), the sperm cell under noises displays a more wavy trajectory and hence requires a longer time to reach the egg (Fig. 7b). The path curvature also illustrates a noisier pattern (Fig. 7c).

**Fig. 8:**
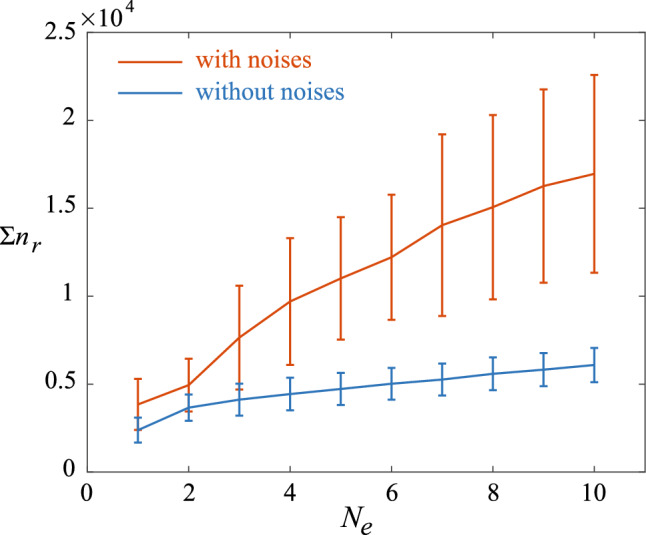
Comparison of navigation performance with and without noises. $\sum n_r$ is the cumulative learning steps required for a 2D sperm cell to reach the egg over increased $N_e$. 10 simulations were performed for each $N_e$. The error bars represent the standard errors of the mean. The red colored line denotes learning with noises and the blue colored line denotes learning without noises. Parameters used: $\kappa _0=0.03$ $\upmu \hbox {m}^{-1}$, $\delta \kappa = 0.006$ $\upmu \hbox {m}^{-1}$, $\delta t =0.75$ s, $v=120$ $\upmu$m/s, $\textbf{x}(t=0)=(360\hat{x}-360\hat{y})$ $\upmu$m, $\lambda =10$ pM$^{-1}$ s$^{-1}$, $\sigma _\kappa =0.005$, $\beta =1/(c_0 \lambda )$. For the cases with noises, $\lambda =10$ pM$^{-1}$ s$^{-1}$, $\sigma _\kappa =0.005$, $\beta =1/(c_0 \lambda )$

We further compare the navigation performance of the cell with and without noises. To this end, we measure the number of learning steps $n_r$ required for the sperm cells to reach the egg and circles around the egg stably thereafter with a radius smaller than 50 $\upmu$m. The time required for the sperm cells reaching the egg is given by $n_r \delta t$. During the learning process, we terminate an episode whenever the cell reaches the threshold of $d<50$ $\upmu$m. Since the navigation strategy obtained by the cell depends on its previous learning experience, $n_r$ in subsequent episodes will be affected by the length of previous episodes. Therefore, we use the cumulative learning steps $\sum n_r$, i.e., the total number of learning steps experienced by the cell, required to reach the egg in each episode for comparison of performance. Figure 8 shows that $\sum n_r$ for cells with noises increases at a faster rate than those cells without noises, hence requiring more learning steps for the reinforcement learning algorithm to obtain converged chemotaxis strategies. Nevertheless, the reinforcement learning algorithm is able to achieve a robust navigation performance via episode learning in the presence of noises.

### Extension to 3D chemotaxis

Finally, we consider the 3D extension of our reinforcement learning approach. The sperm cell model can be extended to 3D by including a binormal vector $\textbf{b}=\textbf{t} \times \textbf{n}$ and a torsion parameter $\tau$ [16]. In this case, the dynamics of the sperm cell is governed by the 3D Frenet-Serret equations:

6 $$\begin{aligned} \begin{aligned} \dot{\textbf{x}}&=v\textbf{t}, \\ \dot{\textbf{t}}&=v\kappa \textbf{n}, \\ \dot{\textbf{n}}&=-v\kappa \textbf{t} + v \tau \textbf{b}, \\ \dot{\textbf{b}}&=-v\tau \textbf{n}. \end{aligned} \end{aligned}$$

The 3D model displays the helical trajectory of the swimming sperm cell, where the curvature and the pitch of the helical path can be controlled by adjusting $\kappa$ and $\tau$. In contrast to the 2D case where the trajectory of the cell forms a closed circular loop when $\kappa$ keeps constant, in the 3D case the cell swims in a helical path with a net translation when $\kappa$ and $\tau$ are constant.

To account for the additional parameters in the 3D model, the state space and the action space of the reinforcement learning algorithm are modified accordingly. In the 3D model, the state of the reinforcement learning agent is specified by the sign for the change in the local chemical field $sgn(\Delta c_n)$, the local curvature $\kappa _n$ and the torsion of the path $\tau _n$. Similar to $\kappa$, $\tau$ is mapped into a set of $L=2X+1$ discrete states with the interval $[\tau _0-X \delta \tau , \tau _0+X \delta \tau ]$, where $\tau _0$ is the initial value for $\tau$ at $n=0$ and $\delta \tau$ is the difference in $\tau$ between two consecutive states. There are two choices of action space corresponding to modulate $\kappa$ and $\tau$ in phase or out of phase, with the same sign for $\delta \kappa$ and $\delta \tau$ or the opposite sign for $\delta \kappa$ and $\delta \tau$, respectively. The action space of the sperm cell includes adding $\kappa _n$ and $\tau _n$ by $\delta \kappa$ and $\delta \tau$, deducting $\kappa _n$ and $\tau _n$ by $\delta \kappa$ and $\delta \tau$, and keeping $\kappa _n$ and $\tau _n$ unchanged. We note that it is possible to treat the variations of $\kappa$ and $\tau$ in each learning step separately, but the learning speed will be reduced due to the larger action space.

**Fig. 9:**
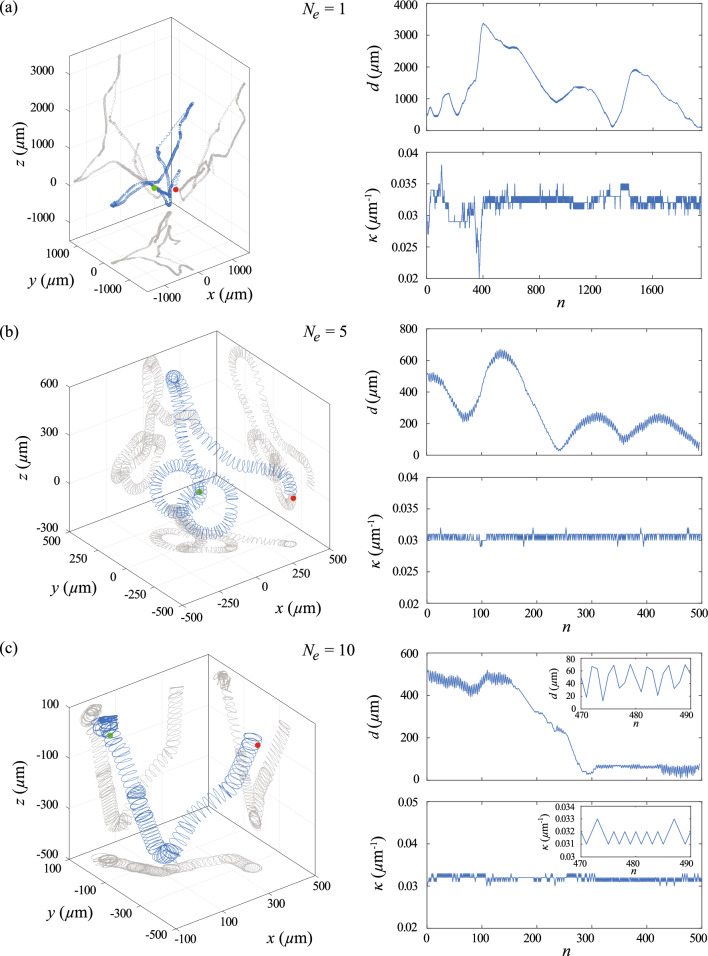
Reinforcement learning approach can be extended to chemotactic navigation in 3D. Trajectory and variation in $\kappa$ of the cell at **a** $N_e=1$, **b** $N_e=5$, **c** $N_e=10$. The insets in (**c**) illustrate the variation of *d* and $\kappa$ of the converged strategy when the cell reaches the egg and stably orbits around it. Parameters used: $\kappa _0 =0.03$ $\upmu \hbox {m}^{-1}$, $\tau _0=0.006$ $\upmu \hbox {m}^{-1}$, $\delta \kappa = 0.001$ $\upmu \hbox {m}^{-1}$, $\delta \tau = -0.004$ $\upmu \hbox {m}^{-1}$, $\delta t =0.5$ s, $v=120$ $\upmu$m/s, $\textbf{x}(t=0)=(360\hat{x}-360\hat{y}+0\hat{z})$ $\upmu$m

The overall learning performance in the 3D cases is similar to the 2D cases. During the learning process (Fig. 9a), the cell initially swims in a random trajectory to explore the surrounding chemical field. Through exploiting the information obtained from the detected chemical field, the reinforcement learning algorithm eventually obtains an effective navigation strategy and determines how the sperm cell modulates the curvature and the torsion of the helical trajectory according to the detected chemical field. The cell varies its $\kappa$ and $\tau$ and slowly steers its helical trajectory toward the egg. The cell finally orbits around the egg at a stable distance of $d \le 50$ $\upmu$m.

Similar to the 2D case, the navigation performance can be significantly enhanced via episode learning (Fig. 9). Less exploration is required by the sperm cell when $N_e$ increases. The cell effectively steers its helical trajectory and smoothly swims toward the egg when $N_e$ is sufficiently large. Interestingly, the converged chemotaxis strategies obtained in 3D reaches the egg with a time similar to the 2D cases, as can be seen from $n_r$ at $N_e=10$, i.e., $n_r=265 \pm 45$ (MEAN and SEM) for the cases in 2D and $n_r=73 \pm 24$ (MEAN and SEM) for the cases of in-phase modulation of $\kappa$ and $\tau$ in 3D and $n_r=188 \pm 97$ (MEAN and SEM) for the cases of out-of-phase modulation, where 10 sets of simulations are performed for each case. We remark that previous studies have demonstrated that optimal chemotaxis is given by an out-of-phase modulation of $\kappa$ and $\tau$ (i.e., opposite sign for $\delta \kappa$ and $\delta \tau$) [6]. However, we do not observe any significant difference in $n_r$ of the converged chemotaxis strategies for both cases. Nevertheless, our result demonstrates the strong potential of the reinforcement learning approach to extend to more complex navigation problems.

## Discussion

In this work, we demonstrate the use of reinforcement learning to mimic navigation strategies for the model of biological cells. Instead of following a biophysical model with an explicit stimulus–response relationship, the reinforcement learning algorithm obtains a policy to modulate key biophysical parameters that control the response to environmental stimuli. As an generic example, we consider a model of sperm cell to illustrate this alternative approach and set a benchmark for future applications of this approach in other biological cells or bioinspired robotics. In particular, helical swimming is a stereotypical behavior adopted by sperm cells and many other microswimmers (e.g., *Euglena gracilis*, *Chlamydomonas reinhardtii*) for their navigation [11, 13, 14]. We anticipate that the approach can be readily extended to other biological cells with helical swimming behaviors which may share similar navigation strategies as the current sperm model.

The chemotaxis strategy of sperm cell obtained by our reinforcement learning algorithm is reminiscent to experimental observations and other models [16], featured by a nearly periodic oscillation in the path curvature which steers the cells toward the egg. Our reinforcement learning approach is robust to sensory and curvature noises, and is readily extensible to 3D. A deeper comparison between the chemotaxis strategies obtained by our reinforcement learning approach and the strategies obtained by other models will be pursued elsewhere. We would like to reiterate that we do not attempt to achieve a realistic model of chemotaxis for biological cells in this work, but rather develop a theoretical framework for decision-making process in biologically relevant navigation based on reinforcement learning. Although it remains questionable whether real biological cells follow a decision-making process similar to reinforcement learning with their chemotactic sensor-actuator network, our approach offers new tools to investigate possible variations of biophysical parameters and the necessary complexity of the sensory system required for developing navigation strategies or more complex biological responses.

We finally discuss several limitations of the current approach and provide possible directions for subsequent investigations. For instance, we only account for the chemical signal released by the egg for simplicity and neglect the effects of physical boundary of the egg as well as the corresponding physical interactions. This can be improved by considering the long-range hydrodynamic interactions and the short-range steric interactions between the motile cells and the egg [46]. The effectiveness of the reinforcement learning approach in navigation problems under fluid shear is also another interesting research question to answer [47–49]. Another possible extension of the current work is to implement the deep neural network with more degrees of freedom that enables the consideration of more biophysical parameters and the handling of continuous spatiotemporal data [27]. Taken together, our reinforcement learning approach provides an alternative avenue for investigating navigation strategies of motile cells and bio-inspired robotics.

## Data Availability

The data supporting the findings of this study are available from the corresponding author upon reasonable request.
